# Tumor Lysis Syndrome Due to Targeting of Hepatocellular Carcinoma Associated with Chronic Myelomonocytic Leukemia

**DOI:** 10.4274/tjh.galenos.2019.2019.0113

**Published:** 2019-08-02

**Authors:** Müfide Okay, Sıla Çetik, İbrahim C. Haznedaroğlu

**Affiliations:** 1 Hacettepe University Faculty of Medicine, Department of Internal Medicine, Division of Hematology, Ankara, Turkey; 2 Hacettepe University Faculty of Medicine, Department of Internal Medicine, Ankara, Turkey

**Keywords:** Leukemia, Myelomonocytic, Chronic, Tumor lysis syndrome, Hematologic neoplasms

## To the Editor,

Targeting hepatic tumors through locoregional application is feasible for anti-tumoral management [1]. Transarterial chemoembolization (TACE) aims to localize chemotherapeutic drugs solely to the tumor, avoiding systemic toxicities [2]. However, the co-existence of hematological malignancies may adversely affect that aim. We would like to point out systemic medical risks by sharing our TACE experience in a patient with hepatocellular carcinoma (HCC) and chronic myelomonocytic leukemia (CMML).

**Figure 1:**
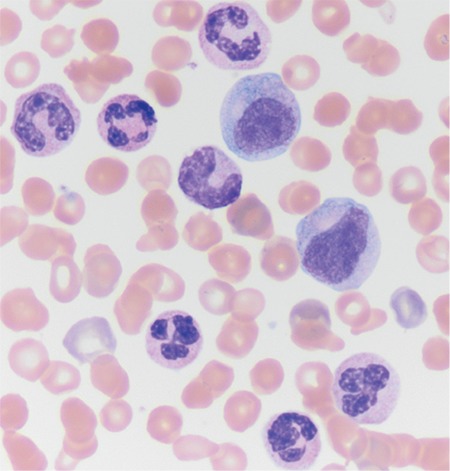
Peripheral blood smear of the patient.

**Figure 2:**
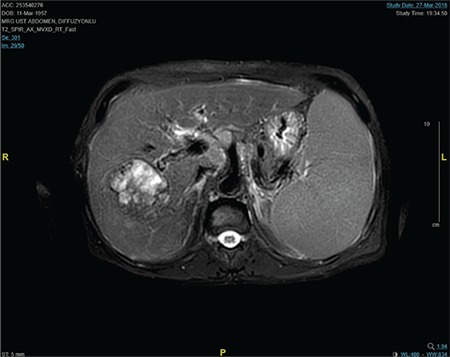
Hepatic tumor in abdominal magnetic resonance imaging.

A 61-year-old male patient with a past medical history of CMML was admitted to our hospital with the findings of right upper quadrant pain and hepatosplenomegaly. Physical examination revealed hepatosplenomegaly compatible with extramedullary hematopoiesis. The clinical presentation was CMML based on the presence of persistent monocytosis, leukocytosis, and dysplastic circulating cells (Figure 1). Although the cytogenetic results revealed a normal karyotype, detailed histopathological bone marrow examination clearly demonstrated CMML with the usual nature of clonality. Four cycles of azacytidine epigenetic therapy were administered immediately after the diagnosis of CMML. The patient was positive for hepatitis B surface antigen and the hepatitis B viral load was high. A diagnosis of chronic hepatitis B infection was reached with histopathological confirmation (fibrosis and hepatitis). Upon admission, his laboratory tests were as follows: alanine aminotransferase, 64 U/L; aspartate aminotransferase, 53 U/L; alkaline phosphatase, 190 U/L; and gamma glutamyl transferase, 162 U/L. Hepatobiliary ultrasound disclosed hypoechoic lesions of 86x66 mm and 67x55 mm with necrosis in the right lobe of the liver. Abdominal magnetic resonance imaging revealed two heterogeneous mass lesions (5.3 cm and 5 cm, respectively) (Figure 2). In the histopathological examination of the liver, HCC was detected. Liver biopsy also showed increments in CD34-positive cells and extramedullary hematopoiesis, consistent with CMML. The patient was diagnosed with HCC, which was classified as stage B according to the Barcelona Clinic Cancer staging. Transarterial ethanol and lipiodol embolization (TACE) therapy was done for tumor ablation. Before TACE, his laboratory tests were as follows: leukocytes, 24.5x10^3^/µL; hemoglobin, 7.9 g/dL; absolute neutrophil count, 15.6x10^3^/µL; platelets, 100x10^3^/ µL; creatinine, 1.18 mg/dL; lactate dehydrogenase, 240 U/L; uric acid, 6 mg/dL; calcium, 8.3 md/dL; potassium, 4 mEq/L; and phosphorus, 4.3 mg/dL. During clinical follow-up, two weeks after the procedure, biochemical studies revealed acute renal failure. Renal function tests were as follows: creatinine, 6.3 mg/dL; phosphorus, 7.1 mg/dL; potassium, 4.7 mEq/L; calcium, 8.8 mg/dL; and uric acid, 30.6 mg/dL. Tumor lysis syndrome was suspected and the patient was hospitalized. Supportive intravenous hydration was started immediately. Allopurinol was initiated at 300 mg twice a day. After two days, his urine output decreased below 100 mL/day and hemodialysis was started. Even though uric acid levels decreased to 7 mg/dL, the patient remained anuric. His clinical condition deteriorated and he developed respiratory distress caused by hemothorax. The complication of hemothorax was ascribed to hemorrhagic diathesis/leukostasis of CMML. The patient was lost due to those systemic complications after 10 days of treatment. Metabolic complications of the patient were ascribed to the TACE procedure during clinical follow-up. The patient signed the informed consent form for sharing patient information.

Chronic myeloid neoplasms of the elderly are associated with poor prognosis [3]. CMML and solid tumors can be observed concurrently [4]. HCC is a malignant tumor due to infection of hepatitis B virus and hepatitis C virus [5]. In our patient, there was hepatitis B positivity and CMML. Moreover, neoplastic CD34-positive cells and malignant tumor cells in the liver microenvironment were striking findings of this case. This is the first such co-existence established in the published literature. TACE is a targeting therapy for HCC lesions with numerous complications [6]. Clinicians should be aware of the fact that targeting tumors can not only cause “local” complications but also could generate systemic adverse medical events such as tumor lysis syndrome and related metabolic disorders, especially in cases of the existence of hematological malignancies [7].
